# Hypermethylation-mediated reduction of WWOX expression in intraductal papillary mucinous neoplasms of the pancreas

**DOI:** 10.1038/sj.bjc.6604986

**Published:** 2009-04-07

**Authors:** S Nakayama, S Semba, N Maeda, M Matsushita, Y Kuroda, H Yokozaki

**Affiliations:** 1 Division of Pathology, Department of Pathology, Kobe University Graduate School of Medicine, Kobe, Japan; 2 Division of Gastroenterological Surgery, Department of Surgery, Kobe University Graduate School of Medicine, Kobe, Japan

**Keywords:** WW domain-containing oxidoreductase, SMAD4, intraductal papillary mucinous neoplasm of the pancreas, hypermethylation

## Abstract

We have previously shown that WW domain-containing oxidoreductase (WWOX) has tumour-suppressing effects and that its expression is frequently reduced in pancreatic carcinoma. In this study, we examined WWOX expression in intraductal papillary mucinous neoplasm of the pancreas (IPMN) to assess the function of WWOX in pancreatic duct tumourigenesis using immunohistochemistry and methylation-specific polymerase chain reaction analysis. Among 41 IPMNs including intraductal papillary mucinous adenomas (IPMAs) and intraductal papillary mucinous carcinomas (IPMCs), loss or reduced WWOX immunoreactivity was detected in 3 (15%) of 20 IPMAs and 17 (81%) of 21 IPMCs. In addition, hypermethylation of the *WWOX* regulatory site was detected in 1 (33%) of 3 WWOX(−) IPMAs and 9 (53%) of 17 WWOX(−) IPMCs, suggesting that hypermethylation may possibly be important in the suppression of WWOX expression. Reduction of WWOX expression was significantly correlated with a higher Ki-67 labelling index but was not correlated with the ssDNA apoptotic body index. Interestingly, decreased WWOX expression was significantly correlated with loss of SMAD4 expression in these IPMNs. The results indicate that downregulation of WWOX expression by the *WWOX* regulatory region hypermethylation is critical for transformation of pancreatic duct.

Intraductal papillary mucinous neoplasm of the pancreas (IPMN) accounts for an estimated 1–3% of all pancreatic exocrine tumours and this pancreatic duct-derived neoplasm is characterised by abundant mucin production, dilated ducts and a more favourable prognosis than that in invasive ductal carcinoma of the pancreas (IDC; Japan Pancreas Society, 2003; Hruban *et al*, 2004). Histologically, intraductal components of IPMN are classified into adenoma (intraductal papillary mucinous adenoma, IPMA) and carcinoma *in situ* (intraductal papillary mucinous carcinoma, IPMC). Interestingly, some patients occasionally have IDC derived from IPMN, suggesting that IPMN corresponds to a precursor lesion, which may potentially progress to IDC (Yamao *et al*, 2000; Sohn *et al*, 2001). Genetic alterations in IPMN have been identified, including mutations in the K*-RAS* (Z’Graggen *et al*, 1997; Schönleben *et al*, 2007), *SMAD4* (Hruban *et al*, 2004), *TP53* (Sessa *et al*, 1994) and *PIK3CA* genes (Schönleben *et al*, 2006), and have been shown to activate the mitogen-activated protein kinase and phosphatidylinositol-3 kinase pathways (Sessa *et al*, 1994; Semba *et al*, 2003). However, much remains unknown about the molecular background of the development and progression of IPMN.

The WW domain-containing oxidoreductase (*WWOX*) gene is a tumour suppressor gene that spans a common chromosomal fragile site, *FRA16D* (16q23.3–24.1). Loss of heterozygosity (LOH) at the *WWOX* locus, hypermethylation of the *WWOX* regulatory site and resultant reduction of WWOX expression have been reported in various human malignancies (Paige *et al*, 2001; Driouch *et al*, 2002; Kuroki *et al*, 2002; Iliopoulos *et al*, 2005). Indeed, restoration of the *WWOX* gene suppresses cell proliferation and induces apoptosis in various human malignancies, including in cells derived from lung cancer (Fabbri *et al*, 2005), prostate cancer (Qin *et al*, 2006) and breast cancer (Aqeilan *et al*, 2007). Recent studies have shown that the WW1 domain of the WWOX protein has an essential function in its tumour suppressor function by regulating the subcellular localisation of p73 (Aqeilan *et al*, 2004a) and AP-2*γ* (Aqeilan *et al*, 2004b), both of which contain the proline-rich ligand ‘PPXY’ motif.

We previously showed that the *WWOX* gene promoter is frequently hypermethylated and WWOX expression frequently reduced in IDC cases (Kuroki *et al*, 2004; Nakayama *et al*, 2008). Much as in other human malignancies, restoration of the *WWOX* gene effectively suppressed the cell growth and induced caspase-dependent apoptosis in pancreatic cancer-derived cells in conjunction with an increase in SMAD4 expression (Nakayama *et al*, 2008). In this study, we assessed WWOX expression in IPMN (IPMA and IPMC) to know whether suppression of WWOX expression is common event during tumourigenesis of these pancreatic duct lesions, IDC and IPMN.

## Materials and methods

### Tissue samples

Formalin-fixed and paraffin-embedded specimens from 41 IPMNs (20 cases of IPMA and 21 cases of IPMC) surgically removed at Kobe University Hospital (Kobe, Japan) were used. Fifteen men and five women (average age, 71.4±7.4 years; age range, 54–82 years) had IPMAs, and eight men and thirteen women (average age, 66.6±8.9 years; age range, 47–80 years) had IPMCs. Informed consent was obtained from all patients and the study was approved by the Kobe University Institutional Review Board. Histological examination was performed according to the Classification of Pancreatic Carcinoma established by the Japan Pancreas Society (2003).

### Immunohistochemistry (IHC)

A modified version of the immunoglobulin enzyme bridge technique with an LSAB kit (Dako, Glostrup, Denmark) was used. Deparaffinised and rehydrated sections were autoclaved to retrieve antigenicity. After blocking of endogenous peroxidase and non-specific reactions, the primary antibodies against WWOX (Imgenex, San Diego, CA, USA), SMAD4 (Cell Signaling, Beverly, MA, USA), Ki-67 (Dako) and ssDNA (Dako) were applied to sections, which were then incubated with biotinylated monkey anti-rabbit IgG. Streptavidin conjugated to horseradish peroxidase was used for the immersion in with 3,3-diaminobenzidine. Sections were counterstained with hematoxylin.

The degree of WWOX and SMAD4 expression was graded according to the number of stained cells and the staining intensity in individual cells: negative, almost no positive cells or <50% of tumour cells showed weak immunoreactivity; positive, >50% of tumour cells showed weak immunoreactivity or tumour cells showed intense immunoreactivity. The Ki-67 labelling index (LI) was determined by counting the positive cells in a total of 1000 tumour cells, whereas the ssDNA apoptotic body index (ssDNA ABI) was determined by counting the ssDNA-positive cells in 10 high-power fields. Detection of ssDNA-positive cells is useful for evaluation of apoptotic tumour cells, as is the terminal deoxynucleotidyl transferase-mediated deoxyuridine triphosphate-biotin nick end-labelling method (Naruse *et al*, 1994; Kawarada *et al*, 1998; Watanabe *et al*, 1999).

### Statistical analysis

We used the *χ*^2^-test and Mann–Whiney *U*-test to evaluate the relationship between WWOX immunoreactivity and the clinicopathological findings. *P*-values less than 0.05 were considered statistically significant.

### Cell culture and treatment

Human pancreatic cancer cell line BxPC-3 was obtained from the American Type Culture Collection (Manassas, VA, USA). Cells were cultured in RPMI 1640 medium containing 10% fetal bovine serum at 37°C in an atmosphere containing 5% CO_2_. BxPC-3 cells were seeded in a 100 mm plate at a density of 1 × 10^6^ cells. After 24 h, cells were treated with DNA methyltransferase inhibitor 5-aza-2′-deoxycytidine (5-aza-dC; Sigma-Aldrich, St Louis, MO, USA) and histone deacetylase inhibitor trichostatin A (TSA; Sigma-Aldrich), either alone or in combination. The medium was changed every day for 5 days. For the treatment combining 5-aza-dC and TSA, cells were cultured in the presence of 5-aza-dC (0–5 *μ*M) for 4 days and then treated with TSA (1 *μ*M) for another 24 h.

### Methylation-specific polymerase chain reaction (MSP) analysis

Genomic DNA was treated with sodium bisulphite (EpiTect Bisulfite kit; Qiagen, Hilden, Germany) and was analysed by MSP using primer sets located within the *WWOX* regulatory site (Iliopoulos *et al*, 2005) for MSP (methylated), 5′-GCGAGTGGATTCGGTAGCGGGCGA-3′ and 5′-CCGTATCGTCCAACCCCGCGT-3′; and for MSP (unmethylated), 5′-GTGAGTGGATTTGGTAGTGGGTGA-3′ and 5′-CCATATCATCCAACCCCACAT-3′. The PCR amplification consisted of 35 cycles (denaturation at 94°C for 30 s, annealing at 62°C for 30 s and extension at 72°C for 1 min) and was followed by a final extension for 10 min at 72°C. The PCR products were resolved by electrophoresis on a 2% agarose gel.

### Bisulphite genomic sequence analysis

Bisulphite sequence analysis was performed to check the methylation status in BxPC-3 cells and IPMN cases. The extracted genomic DNAs were subjected to bisulphite modification and amplification of the 5′ region. The primers were designed from regions in which there are no CpG dinucleotides: 5′-TAAACTATACAAAATCCCAAAT-3′ and 5′-GTTTTTGTAGGATTGGTTAGAA-3′ (Iliopoulos *et al*, 2005). The PCR products were gel-purified using a QIAquick Gel Extraction kit (Qiagen) according to the manufacturer's instructions. Each amplified DNA sample was subcloned and applied to the ABI 310 DNA analyser using a BigDye Terminator kit (Applied Biosystems, Foster City, CA, USA).

### Western blotting

Cells were lysed in a lysis buffer (50 mM Tris-HCl (pH 7.4), 125 mM NaCl, 0.1% Triton X and 5 mM ethylenediaminetetraacetic acid (EDTA)). Proteins (20 *μ*g) were separated by sodium dodecyl sulphate-polyacrylamide gel electrophoresis, electrotransferred onto an immunobilon-P membrane (Millipore, Billerica, MA, USA), and then immunoblotted with antibodies against WWOX and *β*-actin (Sigma-Aldrich). Horseradish-peroxidase-conjugated donkey anti-mouse IgG and sheep anti-rabbit IgG (GE Healthcare, Piscataway, NJ, USA) were used as secondary antibodies. Proteins were visualised using enhanced chemiluminescence.

## Results

### WWOX expression in IPMN and correlation with clinicopathological findings

**Figure 1:**
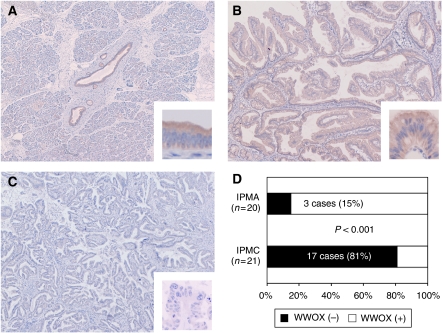
Immunohistochemistry (IHC) of WWOX expression. The degree of WWOX expression was graded according to the number of stained cells and the staining intensity in individual cells: negative, almost no positive cells or <50% of tumour cells showed weak immunoreactivity; positive, >50% of tumour cells showed weak immunoreactivity or tumour cells showed intense immunoreactivity. (**A**) Normal pancreatic ducts (original magnification × 100). (**B**) Intraductal papillary mucinous adenoma (IPMA, original magnification × 100). (**C**) Intraductal papillary mucinous carcinoma (IPMC, original magnification × 100). Each specimen with high magnification ( × 400) is also shown in insets. (**D**) Frequency of reduced or loss of WWOX expression in IPMA and IPMC.

As a first step towards clarifying the function of WWOX in IPMN, we performed immunohistochemical analysis of WWOX expression in 41 IPMN cases. As in the adjacent normal pancreatic duct epithelium, abundant cytoplasmic WWOX expression was detected in IPMA, whereas WWOX expression was remarkably reduced in IPMC. Reduction or loss of WWOX expression was detected in 3 (15%) of 20 IPMAs and 17 (85%) of 21 IPMCs (*P*<0.001; Figure 1A).

Because the malignant potentials of these two subtypes of IPMN, IPMA and IPMC, are quite different, their associations with the clinicopathological findings were evaluated separately. The results are summarised in Table 1. Negative immunoreactivity of WWOX was significantly correlated with greater dilation of the main duct (*P*=0.006) in IPMAs and with older age (*P*=0.022) in IPMCs. Restoration of the *WWOX* gene in pancreatic carcinoma PANC-1 (WWOX-negative) cells effectively suppressed cell growth and induced caspase-dependent apoptosis; we therefore estimated the possible relationship between low levels of WWOX expression and higher growth rate or infrequent incidence of apoptosis in IPMN. The Ki-67 LI was significantly higher in WWOX(−) IPMAs than WWOX(+) IPMAs (*P*<0.001), whereas no correlation was found between the Ki-67 LI and WWOX expression status in IPMCs (*P*=0.109). No difference was found in the ssDNA ABI of these IPMNs between the presence and absence of WWOX expression.

### Hypermethylation of the *WWOX* regulatory site in IPMN

**Figure 2:**
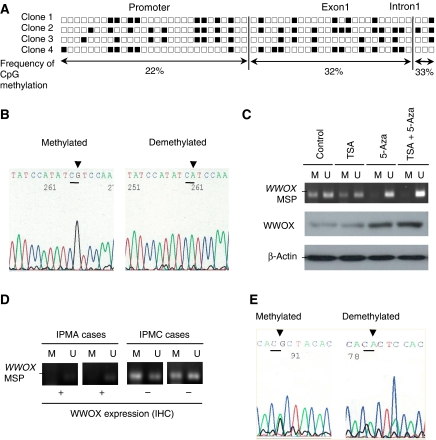
Hypermethylation-mediated downregulation of WWOX expression in intraductal papillary mucinous neoplasm of the pancreas (IPMN). (**A**) Results of bisulphite genomic sequence analysis at the *WWOX* regulatory site, including the promoter region, exon 1 and intron 1. Filled boxes indicate methylation; open boxes indicate absence of methylation. (**B**) Representative results of bisulphite genomic sequence analysis of BxPC-3 cells. Arrowheads indicate the CpG island that demonstrated cytosine–uracil transition. (**C**) The methylation status of the *WWOX* regulatory CpG site determined by the methylation-specific PCR (MSP) analysis. BxPC-3 cells were treated with trichostatin A (TSA), 5-aza-2′-deoxycytidine (5-aza-dC) and a combination of the two drugs. Restored WWOX expression was confirmed by western blot analysis. *β*-Actin was used as a loading control. (**D**) Representative results of MSP analysis. The status of WWOX expression detected by IHC was also exhibited. (**E**) Representative results of bisulphite genomic sequence analysis of IPMA and IPMC. Arrowheads indicate the CpG island that demonstrated cytosine–uracil transition. M, methylated; U, unmethylated.

In pancreatic carcinoma cells and IDC tissue samples, a close correlation has been documented between hypermethylation of the *WWOX* regulatory region and low WWOX expression levels rather than LOH at the *WWOX* locus (Nakayama *et al*, 2008). In BxPC-3 (low WWOX expression) cells, frequent hypermethylation at the *WWOX* regulatory sites was detected by bisulphite sequencing (Figure 2A). Representative results of cytosine–uracil (C-U) transition of the *WWOX* regulatory site are shown in Figure 2B. We confirmed that treatment with the demethylating agent 5-aza-dC led to the restoration of WWOX expression at the protein level (Figure 2C). We then designed an MSP primer set within the *WWOX* regulatory region and investigated the incidence of hypermethylation-mediated suppression of WWOX expression in IPMN. Among 20 IPMNs with low WWOX expression, hypermethylation of the *WWOX* regulatory site was detected in 1 (33%) of 3 WWOX(–) IPMAs and 9 of 17 (53%) of WWOX(–) IPMCs (Figures 2D and E; Table 2).

### Association of SMAD4 expression with the WWOX status in IPMN

**Figure 3:**
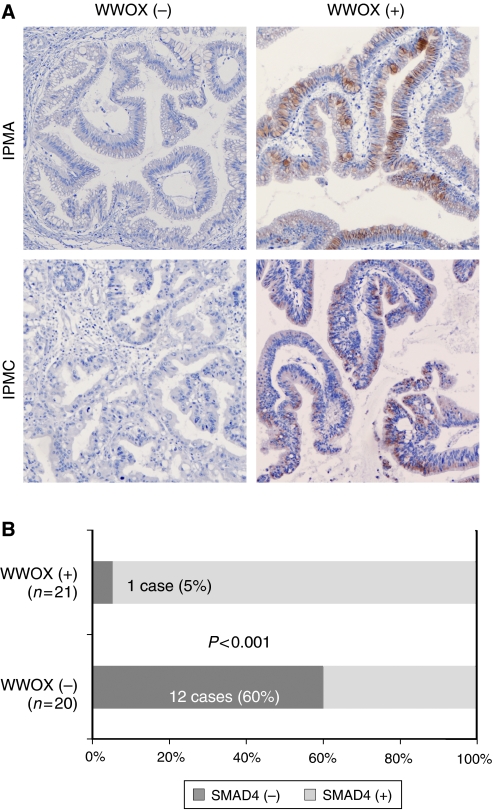
Reduced SMAD4 expression is associated with the WWOX status in IPMN. The degree of SMAD4 expression was graded according to the number of stained cells and the staining intensity in individual cells: negative, almost no positive cells or <50% of tumour cells showed weak immunoreactivity; positive, >50% of tumour cells showed weak immunoreactivity or tumour cells showed intense immunoreactivity. (**A**) Representative IHC results of SMAD4 expression in WWOX(+) and WWOX(−) IPMNs (original magnification × 100). (**B**) Frequency of reduced or loss of SMAD4 expression in WWOX(+) and WWOX(−) IPMNs.

Because we have previously shown that increased SMAD4 expression at the protein level can be induced by the transfection of *WWOX*-expressing vector into pancreatic cancer-derived PANC-1 cells (Nakayama *et al*, 2008), we also examined the SMAD4 expression in these IPMN cases (Figure 3A). In total, 13 (32%) of 41 IPMNs demonstrated loss of SMAD4 expression. Interestingly, loss of SMAD4 expression was detected only in 1 (5%) of 21 WWOX(+) IPMNs, whereas 12 (60%) of 20 WWOX(–) IPMNs showed loss of SMAD4 expression (*P*<0.001; Figure 3B).

## Discussion

Intraductal papillary mucinous neoplasm of the pancreas is one of the unusual neoplasms derived from pancreatic duct epithelia and demonstrates both unusually favourable biological behaviour and a broad spectrum of cytoarchitectural atypia. In this study, we found frequent reduction of WWOX expression in IPMC, which was almost equivalent to the frequency of WWOX reduction in IDC (Nakayama *et al*, 2008). In turn, WWOX expression was relatively retained in most IPMAs, suggesting that reduced WWOX expression does not contribute to development of IPMA. In our previous study, we also reported that the expression of WWOX in pancreatic intraepithelial neoplasias (PanINs) tended to be suppressed in accord with the PanIN grade, which is determined by the morphological and cytogenetic cellular atypia (Nakayama *et al*, 2008). As in the case of other oncogenes (K-*RAS*) and tumour suppressor genes (*p16*, *p53* and *SMAD4*), downmodulation of WWOX expression may be important during multi-step pancreatic duct carcinogenesis, particularly at the late stage (Hahn *et al*, 1996; Hruban *et al*, 2000).

Carcinoma cell lines and primary tumours exhibit hemizygous or homozygous deletion with end points within fragile regions of the human genome, particularly within the most active common fragile site, *FRA3B*, encompassed by the *FHIT* gene (Iliopoulos *et al*, 2006). Similarly, for the *FHIT* gene and the other fragile site-related genes, deletion at the *WWOX* (*FRA16D*) locus and resultant loss of WWOX expression have been shown to promote cell transformation and immortalisation in various human malignancies (Paige *et al*, 2001; Driouch *et al*, 2002; Kuroki *et al*, 2002); however, the incidence of LOH was infrequent in pancreatic carcinoma (Kuroki *et al*, 2004; Nakayama *et al*, 2008) and we could not detect LOH in this series of IPMNs (data not shown). Indeed, Fukushige *et al* (1998) did not detect aberrant copy numbers of DNA sequences on the *WWOX* locus (16q23.3–24.1) in pancreatic adenocarcinomas by the comparative genomic hybridisation method. Taken together, these results indicated that hypermethylation at the *WWOX* regulatory site, but not LOH, is a critical for the suppression of *WWOX* transcripts during pancreatic duct carcinogenesis. Recently, Irizarry *et al* (2009) reported that most methylation alterations in colon cancer occur not in promoters, and also not in CpG islands, but in sequences up to 2 kb distant called ‘CpG island shore’, using comprehensive high-throughput array-based relative methylation analysis. In the future, we would like to identify such a tissue-specific conserved region for WWOX expression to explain frequent reduction of WWOX expression in the development of IPMC from IPMA.

Loss of WWOX expression was significantly correlated with Ki-67 LI but not with ssDNA ABI. Also, we noted that reduction of WWOX expression was associated with the status of SMAD4 expression. The *SMAD4* gene (also referred as *DPC4*, for deleted in pancreatic carcinoma locus 4) shares characteristics with typical tumour suppressor genes, including involvement in the transforming growth factor-*β* signalling (Hahn *et al*, 1996). About half of pancreatic carcinomas contain either homozygous deletions of the *SMAD4* locus or inactivating mutations in one allele associated with LOH, and a resultant loss of SMAD4 expression in pancreatic carcinoma has been documented (Hahn *et al*, 1996; Wilentz *et al*, 2000). Takahashi *et al* (2004) investigated the SMAD4 status in the intraductal carcinoma component of IDC and found that 20–59% of carcinomas *in situ* expressed SMAD4, which corresponds to our data obtained in this study. The molecular mechanism underlying the simultaneous negative regulation of WWOX and SMAD4 levels is still unknown; according to our previous results, however, restoration of WWOX expression increased SMAD4 expression at the protein level, but not at the mRNA level, suggesting the WWOX-mediated post-transcriptional regulation of SMAD4 levels (Nakayama *et al*, 2008). SMAD4 can be proteasomally degraded after polyubiquitination by SMURF1 and SMURF2 E3 ligase complexes, which possess the WW domain (Morèn *et al*, 2003, 2005). Hence, we estimate that SMURFs and WWOX may competitively bind to the SMAD4-binding protein SMAD2 and SMAD7, both of which contain the PPXY motif, consequently leading inhibition of SMURF-related SMAD4 degradation. However, further investigation will be necessary to elucidate the mechanism by which SMAD4 levels can be increased by WWOX in IPMN. We assume that downregulation of WWOX is one of the critical events during the progression and development of IPMN, particularly at the late step of malignant transformation of pancreatic duct lesions, in addition to loss of SMAD4 expression.

## Figures and Tables

**Table 1:** Correlation of WWOX expression with clinicopathological findings in IPMA

	**WWOX(−)**	**WWOX(+)**	
**IPMA**	**(*n*=3)**	**(*n*=17)**	***P*-value^a^**
*Age (years)*			
Average	71.0±2.6	69.9±7.9	0.816
			
*Gender*			
Male	2%	13%	0.860
Female	1%	4%	
			
*Tumour size (mm)*			
Average	20.7±12.5	41.0±20.7	0.121
			
*Main duct (mm)*			
Average	12.3±3.1	5.6±3.4	0.006
			
*Location*			
Head	3%	12%	0.399
Body/Tail	0%	5%	
			
*Tumour type*			
Main duct	2%	6%	0.344
Branch	1%	11%	
			
Ki-67 LI^b^	22.3±6.5	5.1±5.1	<0.001
ssDNA ABI^b^	1.7±0.8	1.4±0.9	0.624

IPMA=intraductal papillary mucinous adenoma; Ki-67 LI=Ki-67 labelling index; ssDNA ABI=ssDNA apoptotic body index.

a *P*-value less than 0.05 was considered to be statistically significant.

b Ki-67 LI was determined by counting the positive cells in 1000 tumour cells, whereas the ssDNA ABI was determined by counting the ssDNA-positive cells in 10 high-power fields.

**Table 2:** Correlation of WWOX expression with clinicopathological findings in IPMC

	**WWOX(−)**	**WWOX(+)**	
**IPMC**	**(*n*=17)**	**(*n*=4)**	***P*-value^a^**
*Age (years)*			
Average	68.7±7.6	57.8±9.1	0.022
			
*Gender*			
Male	7%	1%	0.502
Female	10%	3%	
			
*Tumour size (mm)*			
Average	61.9±32.9	50.0±24.5	0.507
			
*Main duct (mm)*			
Average	9.5±3.8	5.8±1.7	0.071
			
*Location*			
Head	12%	3%	0.684
Body/Tail	5%	1%	
			
*Tumour type*			
Main duct	14%	3%	0.852
Branch	3%	1%	
Ki-67 LI^b^	27.6±10.8	14.4±3.3	0.109
ssDNA ABI^b^	1.5±1.2	1.2±0.4	0.767

IPMC=intraductal papillary mucinous carcinoma; Ki-67 LI=Ki-67 labelling index; ssDNA ABI=ssDNA apoptotic body index.

a *P*-value less than 0.05 was considered to be statistically significant.

b Ki-67 LI was determined by counting the positive cells in 1000 tumour cells, whereas the ssDNA ABI was determined by counting the ssDNA-positive cells in 10 high-power fields.
